# Increase in Blood-Brain Barrier (BBB) Permeability Is Regulated by MMP3 via the ERK Signaling Pathway

**DOI:** 10.1155/2021/6655122

**Published:** 2021-03-30

**Authors:** Qin Zhang, Mei Zheng, Cristian E. Betancourt, Lifeng Liu, Albert Sitikov, Nikola Sladojevic, Qiong Zhao, John H. Zhang, James K. Liao, Rongxue Wu

**Affiliations:** ^1^Department of Biological Sciences Division-Cardiology, University of Chicago, USA; ^2^Department of Anesthesiology, Tongji Hospital, Tongji Medical College, Huazhong University of Science and Technology, China; ^3^Division of Cardiology, Department of Medicine, Inova Heart and Vascular Institute, USA; ^4^Center for Neuroscience Research, Loma Linda University, School of Medicine, USA

## Abstract

**Background:**

The blood-brain barrier (BBB) regulates the exchange of molecules between the brain and peripheral blood and is composed primarily of microvascular endothelial cells (BMVECs), which form the lining of cerebral blood vessels and are linked via tight junctions (TJs). The BBB is regulated by components of the extracellular matrix (ECM), and matrix metalloproteinase 3 (MMP3) remodels the ECM's basal lamina, which forms part of the BBB. Oxidative stress is implicated in activation of MMPs and impaired BBB. Thus, we investigated whether MMP3 modulates BBB permeability.

**Methods:**

Experiments included *in vivo* assessments of isoflurane anesthesia and dye extravasation from brain in wild-type (WT) and MMP3-deficient (MMP3-KO) mice, as well as *in vitro* assessments of the integrity of monolayers of WT and MMP3-KO BMVECs and the expression of junction proteins.

**Results:**

Compared to WT mice, measurements of isoflurane usage and anesthesia induction time were higher in MMP3-KO mice and lower in WT that had been treated with MMP3 (WT+MMP3), while anesthesia emergence times were shorter in MMP3-KO mice and longer in WT+MMP3 mice than in WT. Extravasation of systemically administered dyes was also lower in MMP3-KO mouse brains and higher in WT+MMP3 mouse brains, than in the brains of WT mice. The results from both TEER and Transwell assays indicated that MMP3 deficiency (or inhibition) increased, while MMP3 upregulation reduced barrier integrity in either BMVEC or the coculture. MMP3 deficiency also increased the abundance of TJs and VE-cadherin proteins in BMVECs, and the protein abundance declined when MMP3 activity was upregulated in BMVECs, but not when the cells were treated with an inhibitor of extracellular signal related-kinase (ERK).

**Conclusion:**

MMP3 increases BBB permeability following the administration of isoflurane by upregulating the ERK signaling pathway, which subsequently reduces TJ and VE-cadherin proteins in BMVECs.

## 1. Introduction

The blood-brain barrier (BBB) regulates the exchange of molecules between the brain and peripheral blood and is composed primarily of endothelial cells (ECs), as well as pericytes and the endfeet of astrocytes [1]. Brain microvascular endothelial cells (BMVECs) form the lining of cerebral blood vessels and are linked to each other via tight junctions (TJs), and the structural stability of TJs is maintained, in part, by adherens junctions (AJs) [2, 3]. Numerous studies have shown that increases in BBB permeability are associated with the degradation of TJs and AJs in ECs [4–7], which can disrupt brain function [8, 9] by augmenting the passage of macrophages, leukocytes, endotoxins, bacteria, and drugs from the peripheral circulation into the brain [10–12]. Thus, the regulation of BBB permeability is crucial both for protecting the brain from harmful components of the peripheral circulation and for the treatment of central nervous system (CNS) disorders. The BBB is regulated primarily via interactions between its cellular components and the extracellular matrix (ECM); thus, matrix metalloproteinases (MMPs), which are the principal drivers of ECM degradation and remodeling, appear to have a key role in breaking down the BBB. [13]. Furthermore, activation of the extracellular signal-related kinase (ERK) pathway has been shown to induce BBB hyperpermeability by changing the composition of TJs [14–16], but TJ proteins in sheep pleura remained intact after exposure to MMP2 or MMP9 [17, 18], which suggests that these two MMPs regulate BBB permeability via an alternative mechanism. MMP3, another member of the MMP family, is a zinc-dependent protease that is activated through autocleavage, and activated MMP3 remodels the basal lamina of the ECM, which forms part of the BBB [19]. However, whether MMP3 regulates BBB integrity, and if so, whether its role is mediated by ERK signaling and/or changes in TJ stability, remains unclear.

For most molecules, passive permeability of the BBB is low, but inhaled anesthetics such as isoflurane are highly fat soluble and, consequently, can traverse the BBB and enter brain tissue [20]. Nevertheless, factors that increase BBB permeability augment the passage of isoflurane into the CNS and enhance anesthesia [9]. Thus, the experiments presented in this report evaluated the potential role of MMP3 in BBB permeability by combining *in vivo* assessments of isoflurane anesthesia and dye extravasation in wild-type (WT) and MMP3-deficient mice with *in vitro* assessments of the integrity of BMVEC cell layers and the expression of junction proteins. Our results indicate that MMP3 could be therapeutically targeted to manipulate BBB permeability and treat neurological disease.

## 2. Materials and Methods

### 2.1. Animals

All animal procedures were carried out in accordance with the guidelines provided by the Institutional Animal Care and Use Committee of the University of Chicago (Chicago, Illinois). The C57BL/6-MMP3 deficient (MMP3−/−) mice were a GEM Collection Model TTM-610 and were generated as described before [21]. The line was backcrossed for 12 generations with C57BL/6 mice, which were provided by the Taconic Biosciences company. A PCR-based analysis was employed to genotype mice. Mice were housed in a room at 22-24°C on a 12-hour light/dark cycle and received drinking water *ad libitum*. We used 8-week-old male mice in this study.

Genotyping: We isolated genomic DNA from mouse tail clips using the Puregene DNA isolation kit from Gentra Systems according to the manufacturer's instruction. About 10 ng of the genomic DNA was used for PCR.

### 2.2. Experimental Design

**Figure 1:**
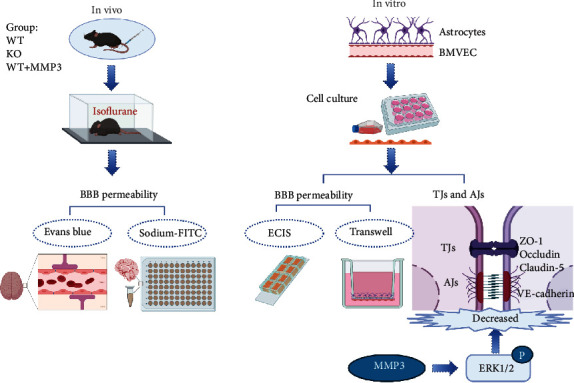
Experimental design. Experiments were conducted both *in vivo* and *in vitro*, as indicated. *In vivo* experiments were conducted in wild-type mice (WT), MMP3-KO mice (KO) mice, and WT mice administered MMP3 (WT + MMP3) and included assessments of susceptibility to isoflurane anesthesia and BBB permeability to intravenously administered dyes (Evans blue, sodium-FITC). *In vitro* assessments included measurements of barrier integrity via TEER using ECIS system in monolayers of BMVECs and in cocultures of BMVECs and astrocytes via Transwell assay. The abundance of junctional proteins was also evaluated via Western blot.

The experimental design of the current study is described below and illustrated in Figure 1. All experiments were conducted in a blinded fashion to investigate the influence of MMP3 on the BBB integrity, we assigned all the mice to three different groups under isoflurane, including WT (MMP3+/+), KO (MMP3-/-), and WT+MMP3 (the mice were administered a recombinant human MMP3 via tail intravenous injection (iv), which was purchased from Abcam). Each experimental group contained 8 mice. *In vivo*, the BBB permeability was evaluated by measuring Evans blue and sodium-FITC-dextrans extravasation [22]. In vitro study, the BBB permeability was detected in an *in vitro* model of BBB. To evaluate the role of MMP3 on BBB junction proteins, we used the MMP3 recombinant human protein to stimulate brain microvascular endothelial cells (BMVECs). Afterward, we detected the protein level of MMP3, ZO-1, occludin, VE-cadherin, and claudin-5 by Western blot and fluorescence staining. To increase the MMP3 levels, lipopolysaccharide (LPS) in a dose of 100 *μ*g/mL as a stimulus to increase the MMP3 levels [23, 24], and then, the ERK inhibitor FR1080204 [25] (purchased from Sigma-Aldrich) was administered to investigate the mechanism behind the effects of MMP3 on the BBB.

### 2.3. *In Vitro* Model of the Blood-Brain Barrier

To mimic the anatomic structure of the *in vivo* BBB, we developed an *in vitro* BBB model based on the coculture of brain ECs with astrocytes, as described in other publications [26]. In this model, BMVECs were seeded in the Transwell insert, and then, primary isolated astrocytes were grown on the undersurface of the Transwell insert. Isolation of the BMVECs and astrocytes are described in detail in the supplemental file (available here).

### 2.4. MMP3 Administration *In Vivo* and *In Vitro* Study

The MMP3 recombinant human protein purchased from Sigma was diluted to 20 *μ*g/mL in the assay buffer: 50 mM Tris, 10 mM CaCl2, 150 mM NaCl, 0.05% (w/v), and Brij-35, pH 7.5. For the next step, MMP3 was then activated by adding chymotrypsin (Sigma, 1 mg/mL stock in 1 mM HCl) to a final concentration of 5 *μ*g/mL. Afterward, the preparation was incubated at 37°C for 30 minutes [27], and a dose of 50 *μ*g/kg of the MMP3 preparation was injected into the tail vein 30 minutes before an Evans blue injection *in vivo*. The activated MMP3 was added to the *in vitro* cells at a dose of 150 ng/mL for 24 hours.

### 2.5. Isoflurane Exposure

We anesthetized the mice with isoflurane in the chamber until they lost consciousness and respiration showed to an appropriate rate. A typical exposure includes 3% isoflurane and 100% O2, which corresponds to a respiration rate of 1 breath every 2 sec. A response to a toe pinch or tail pinch was used to confirm the appropriate state of anesthesia. The mice were orally intubated with a 22G IV catheter and artificially ventilated with a rodent respirator (Harvard 1. Apparatus or similar device, tidal volume 0.3 mL, rate 105 strokes/min) [28]. The air from the thorax was evacuated through a tub, which was connected from the mice noses and extended to a Fluosorber canister. Anesthesia was maintained with 1–2% isoflurane in 100% oxygen for 1 hour. The concentration of isoflurane was adjusted according to the loss and regain of reflexes. During anesthesia, mice were placed on a heating pad to maintain body temperature at 37°C and monitored until fully ambulatory. Anesthesia was terminated by discontinuing isoflurane administration. The anesthetic effect of isoflurane (induction time, emergence time, and isoflurane usage volume) was recorded carefully.

### 2.6. *In Vivo* BBB Permeability Assay in Mice

Either Evans blue [29, 30] or a sodium-Fluorescein Isothiocyanate (FITC) assay [31] was used for the assessment of the BBB integrity. The mice were injected with 2% of Evans blue or sodium-FITC (1 mg/mL in saline) 200 *μ*L through the tail vein 2 hours before sacrificing them. Moreover, they were anesthetized with 1-2% isoflurane for 1 hour and then transcardially perfused with 10 mL normal saline to remove the Evans blue dye and the sodium-FITC from the blood vessels. Brain tissue from the bilateral temporal lobes was carefully removed from the animals following sacrifice. Regarding the mice injected with Evans blue dye, a total of 0.5 mL of formamide was added to the brain tissue homogenate to dissolve the Evans blue dye. After incubation in a 55°C water bath for 48 hours, the samples were centrifuged at 12000 × g for 30 minutes. The supernatant was then collected before absorbance measurements at 632 nm were performed using a UV spectrophotometer (Hitachi, Ltd.). The Evans blue content was calculated according to the standard curve. For mice that were injected with sodium-FITC, a total of 1 mL 50 mM of Tris buffer solution was added to the brain tissue homogenate and then centrifuged at 3000 rpm for 30 minutes. We proceeded to collect the supernatant, which was then mixed with methanol (1 : 1) and centrifuged at 3000 rpm for 30 min. The supernatant was collected, and its fluorescent intensity measured on a plate reader. Finally, the concentration per mg of tissue was calculated using a standard curve.

### 2.7. RNA Isolation, Reverse Transcription, and Quantitative PCR

Total RNA was isolated from cells and tissue using a RNeasy mini kit (Qiagen, Valencia, CA) according to the manufacturer's protocol. The extracted RNA was treated with recombinant DNAse (Invitrogen, Grand Island, NY) according to the manufacturer's protocol to remove any DNA contamination. cDNA was synthesized from 500 ng of RNA using the Superscript III reverse transcription kit (Invitrogen). Quantitative real-time PCR was performed using a 7300 Real-Time PCR System (Applied Biosystems, Foster City, CA) to determine the gene expression for MMP3 using validated TaqMan® gene expression assay primer/probe combinations (Applied Biosystems). All qPCR results were normalized to the expression of the endogenous control 18S. Folding changes in the transcripts were determined using the delta cycle threshold (i.e., ΔΔCt) method.

### 2.8. Transendothelial Electrical Resistance (TEER) Measurement

Measurements of transendothelial electrical resistance (TEER) across the mice brain EC monolayers were performed using the electrical cell-substrate impedance sensing system (ECIS) (Applied BioPhysics, Troy, NY, USA), as described in a previous study [32, 33]. For impedance measurements, cells were grown on 8W10E+ arrays. The arrays were treated with 10 mM L-cysteine (cat#C7352-25G, Sigma-Aldrich) followed by coating with collagen type I (cat#A1048301, Thermo Fisher Scientific) at 1 *μ*g/cm^2^. The arrays of the electrical stabilization command in the ECIS software were used to sterilize and clean the gold electrodes. BMVECs were seeded onto the arrays at a density of 60,000 cells/cm^2^ in 400 *μ*L of L-DMEM growth media. ECIS was conducted using the multiple frequency/time (MFT) option to record the impedance measurements over a broad spectrum of frequencies.

### 2.9. Detection of BBB Permeability *In Vitro* Study

To construct an *in vitro* BBB model [34], we isolated and identified mice BMVECs and astrocytes (Supplementary Figure 1). We placed a Transwell chamber insert with a 0.4-*μ*m aperture (Corning, New York, USA) into volumetric flasks. Astrocytes (1 × 10^6^/mL) were added to the underside of the Transwell and incubated at 37°C and 5% CO_2_ for 24 hours. The chambers were then placed carefully into a 6-well plate (Corning) (Figure 1(b)). When the astrocytes reached 60% confluence under an inverted microscope, ECs (1 × 10^7^/mL) were seeded on top of the Transwell (Figure 1(c)) at 37°C and 5% CO_2_. Furthermore, we observed the cells under an inverted microscope until the astrocytes and BMVECs reached a high-density coculture and, at that point, a FITC-dextran (Dextran-blue-3KD and Dextran-red-40KD) [35] Transwell assay was used to assess the *in vitro* BBB permeability.

### 2.10. Western Blot

Cells were placed on ice, and the media was aspirated prior to washing twice with ice-cold PBS. A cell lysis buffer was prepared by adding a complete mini protease inhibitor tablet (Roche Diagnostics, Indianapolis, IN) and phosphatase inhibitors (Sigma-Aldrich) to a protein isolation buffer (40 mM HEPES, 120 mM NaCl, 1 mM EDTA, 3% CHAPS *w*/*v*). Ice-cold buffer was added to each well, and the cells were mechanically disrupted using a rubber scraper. We performed Western blots, as described before in my paper [36]. Briefly, protein extracts from the cells were subjected to a freeze/thaw cycle before being centrifuged (6,000 rpm ×20 min). Supernatants were collected, protein concentrations were determined using a BCA assay (Thermo Fisher SDS-PAGE was performed using 4–12% Bis/Tris gels (Biorad), and proteins were transferred to nitrocellulose membranes. After blocking with 5% non-fat milk in TBST, we incubated the membrane with anti-MMP3 (1 : 1000; Abcam), anti-ZO-1 (1 : 1000, Invitrogen), anti-occludin (1 : 1000, Invitrogen), anti-VE-cadherin (1 : 500, Invitrogen), anti-claudin-5 (1 : 1000, Invitrogen), and anti-p-ERK (1 : 2000 Invitrogen) antibodies for 16 hours at 4°C. After incubating with a Horseradish peroxidase- (HRP-) conjugated secondary antibody, the membranes were developed with enhanced chemiluminescence (ECL) reagent and imaged with a ChemiDoc imaging system (Biorad). A quantitative assessment was performed using Image Lab software (Biorad).

### 2.11. Assessment of Protein Expression by Quantitative Immunofluorescence

Fixed samples were thawed and rehydrated with PBS containing glycine at 50 mM (Sigma-Aldrich) for 10 minutes. Cells were then permeabilized with 0.1% Triton X-100, and nonspecific binding sites were blocked with a blocking buffer (PBS containing 1% bovine serum albumin BSA and 10% serum from the species the secondary antibodies were raised in). Cells were incubated overnight at 4°C with primary antibodies against one of the following targets: vWF (1 : 500 dilution; Dako, Carpentaria, CA), GFAP (1 : 50 dilution; Invitrogen, Carlsbad, CA), MMP3 (1 : 50 dilution; Invitrogen, Carlsbad, CA), or ZO-1 (1 : 50 dilution; Invitrogen, Carlsbad, CA). After incubation with the primary antibodies, the slides were washed and incubated with fluorescent-labeled secondary antibodies (1 : 200 dilution; Invitrogen, Carlsbad, CA) and mounted under glass coverslips with Vectashield-containing DAPI for nuclear identification (Vector Laboratories, Burlingame, CA). Slides were imaged on a fluorescent microscope and analyzed using Image J software. The average maximum intensity was calculated, and this value was used as the measurement threshold. Next, ten fields were selected at random, and the average of the maximum and mean intensities were calculated correcting for the background threshold. Three images per animal were assessed.

### 2.12. Detection of MMP3 Levels

Both MMP3 serum levels and MMP3 protein expression levels in cultured BMVECs were also determined using a specific ELISA kit by following the manufacturer's instructions (ELISA kit Abcam; ab203363). 50 *μ*L of serum (diluted 100 times) and cell culture supernatants or standards were added together with an antibody cocktail to appropriate wells. After incubating for 1 hour at room temperature on a plate shaker set to 400 rpm, the microplate was washed 3 times. Further, 100 *μ*L of TMB Substrate was added to each well. Finally, the OD was recorded at 450 nm after stopping the reaction by adding 100 *μ*L of Stop Solution.

### 2.13. Statistical Analysis

All data are shown as the mean ± SD. Data were determined to be normal by the Shapiro-Wilks test, and unpaired *t*-tests were used for comparisons between two groups. For comparisons between multiple groups, a one-way ANOVA with Tukey's multiple comparisons test or a two-way ANOVA with Bonferroni post hoc test were performed depending on the number of experimental variables. A two-tailed *p* value of less than 0.05 was considered statistically significant. Data were visualized and analyzed using SPSS version 17.0 (Armonk, NY) and GraphPad Prism version 8.0.0 (San Diego, CA).

## 3. Results

### 3.1. MMP3 Is Highly Expressed in BMVECs

**Figure 2:**
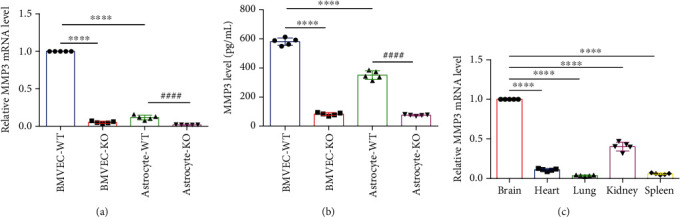
MMP3 is predominantly expressed in BMVECs. (a) MMP3 mRNA abundance was evaluated via q-PCR in BMVECs and astrocytes isolated from the brains of WT (BMVEC-WT and Astrocyte-WT) and MMP3-KO (BMVEC-KO and Astrocyte-KO) mice; results were normalized to measurements in BMVEC-WT. ^∗∗∗∗^*p* < 0.0001, ^####^*p* < 0.0001. (b) MMP3 protein abundance was evaluated via ELISA in culture medium from the indicated cell populations. ^∗∗∗∗^*p* < 0.0001, ^####^*p* < 0.0001. (c) MMP3 mRNA abundance was evaluated in microvascular endothelial cells (MVECs) isolated from the brains, hearts, lungs, kidneys, and spleens of WT mice results were normalized to measurements in brain MVECs. ^∗∗∗∗^*p* < 0.0001.

**Figure 3:**
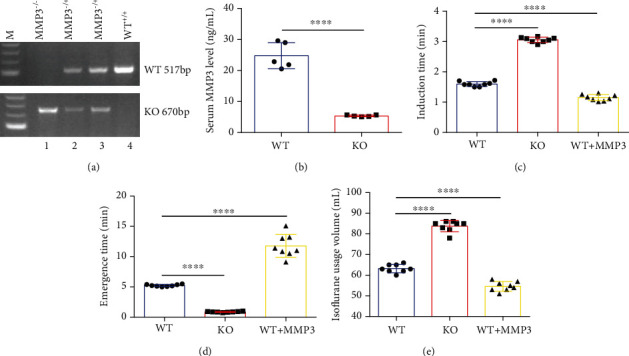
MMP3 enhances the anesthetic effect of isoflurane. (a) The genotypes of WT (WT+/+), heterozygous MMP3-KO (MMP3–/+), and homozygous MMP3-KO (MMP3–/–) mice were confirmed via PCR; the WT and KO alleles were identified with 517-bp and 670-bp fragments, respectively. (b) MMP3 protein abundance was evaluated via ELISA in the serum of WT and homozygous MMP3-KO (KO) mice. ^∗∗∗∗^*p* < 0.0001. (c–e) WT mice, MMP3-KO mice, and WT mice treated with MMP3 were anesthetized with inhaled isoflurane, and the (c) time to the onset of anesthesia, (d) time to emergence from anesthesia, and (e) total volume of isoflurane was recorded. ^∗∗∗∗^*p* < 0.0001.

Measurements of MMP3 mRNA (1 vs. 0.117 ± 0.014, *p* < 0.0001; Figure 2(a)) and protein abundance (581.2 ± 25.11 pg/mL vs. 350.7 ± 29.33 pg/mL, *p* < 0.0001; Figure 2(b)) confirmed that the MMP3-KO mutation dramatically reduced MMP3 levels in BMVECs and astrocytes. However, measurements in WT mice indicated that MMP3 expression was significantly higher in BMVECs than in primary astrocytes or in microvascular endothelial cells (MVECs) isolated from the heart, lung, kidney, or spleen (1 vs. 0.111 ± 0.016 vs. 0.037 ± 0.007 vs. 0.404 ± 0.056 vs. 0.058 ± 0.011, *p* < 0.0001) Figure 2(c)), which suggests that MMP3 may have a unique and important role in BMVECs that differs from its roles in ECs from other organs. Thus, we began to investigate whether MMP3 modulates BBB integrity by monitoring the effect of isoflurane anesthesia in MMP3-KO mice (*n* = 8), in WT mice (*n* = 8) (Figures 3(a) and 3(b)), and in WT mice that had been treated with intravenous injections of recombinant human MMP3 (WT+MMP3; *n* = 8) (bodyweight: 28.73 ± 1.111 g, 28.13 ± 0.75 g, and 28.05 ± 1.09 g, respectively, *n* = 8, *p* = 0.352). Compared to assessments in WT mice, measurements of anesthesia induction time were higher in MMP3-KO mice (3.058 ± 0.088 min vs. 1.598 ± 0.080 min; *p* < 0.0001; Figure 3(c)) and lower in WT+MMP3 mice (1.148 ± 0.104 min vs. 1.598 ± 0.080 min; *p* < 0.0001; Figure 3(c)), anesthesia emergence times were shorter in MMP3-KO mice (0.882 ± 0.073 min vs. 5.233 ± 0.173 min; *p* < 0.0001; Figure 3(d)), and longer in WT + MMP3 mice (11.780 ± 1.883 vs. 5.233 ± 0.173; *p* < 0.0001; Figure 3(d)), and isoflurane usage volumes were larger in MMP3-KO mice (83.750 ± 0.959 mL vs. 63.250 ± 0.700 mL; *p* < 0.0001; Figure 3(e)) and smaller in WT+MMP3 mice (54.630 ± 0.822 mL vs. 63.250 ± 0.700 mL; *p* < 0.0001; Figure 3(e)). Thus, MMP3 appears to increase susceptibility to isoflurane anesthesia.

### 3.2. MMP3 Promotes BBB Permeability in Mice

**Figure 4:**
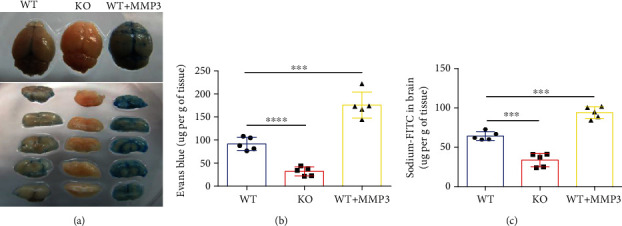
MMP3 increases BBB permeability. (a, b) Evans blue dye was administered via tail-vein intracardial injection to WT mice, MMP3-KO mice (KO), and WT mice that had been treated with MMP3. Two hours later, saline was injected to clear the dye from the vasculature, and the brains were harvested. Extravasation of the dye was evaluated (a) qualitatively in brain images and (b) quantitatively via spectrophotometric measurements of absorbance (632-nm wavelength). Dye quantities were calculated by comparing absorbance measurements to a standard curve. ^∗∗∗∗^*p* < 0.0001, ^∗∗∗^*p* < 0.001. (c) Sodium-FITC was administered via tail-vein injection to mice, and dye extravasation was quantified 2 hours later via fluorescence intensity. Dye quantities were calculated by comparing fluorescence measurements to a standard curve. ^∗∗∗^*p* < 0.001.

To determine whether the relationship between MMP3 expression and isoflurane anesthesia was mediated by changes in BBB permeability, mice were systemically injected with Evans blue dye or sodium-FITC and anesthetized; then, the dyes were cleared from the vessels via saline infusion, and extravasation of the dyes into the brain parenchyma was evaluated [22, 37]. Little evidence of Evans blue extravasation was observed in the brains of MMP3-KO mice, and MMP3 treatment visibly increased the amount of Evans blue in WT mouse brains (Figure 4(a)). Furthermore, both Evans blue (90.796 ± 13.112 *μ*g per gram of tissue vs. 32.009 ± 8.620 *μ*g per gram of tissue; *p* < 0.0001; Figure 4(b)) and sodium-FITC (33.660 ± 8.457 *μ*g per gram of tissue vs. 63.107 ± 6.710 *μ*g per gram of tissue; *p* < 0.001; Figure 4(c)) levels were significantly lower in MMP3-KO mouse brains, and significantly greater in WT+MMP3 mouse brains (180.971 ± 26.829 *μ*g per gram of tissue vs. 90.796 ± 13.112 *μ*g per gram of tissue; *p* < 0.001; 94.111 ± 7.464 *μ*g per gram of tissue vs. 63.107 ± 6.710 *μ*g per gram of tissue; *p* < 0.001; Figures 4(b) and 4(c)), than in the brains of WT mice. Collectively, these observations suggest that MMP3 may regulate BBB permeability.

### 3.3. MMP3 Regulates the Integrity of BMVEC Monolayers *In Vitro*

**Figure 5:**
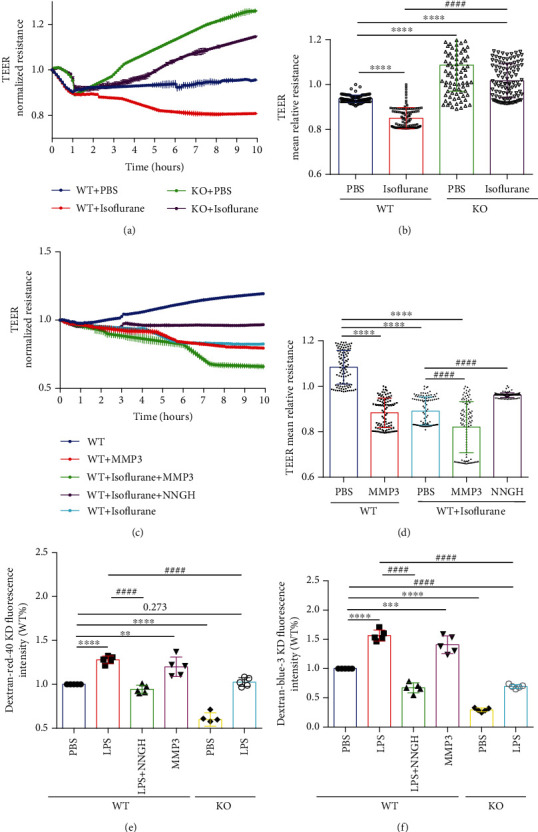
MMP3 reduces the integrity of BMVEC monolayers. (a, c) TEER measurements were recorded in monolayers of WT and MMP3-KO (KO) BMVECs during treatment with the indicated combinations of phosphate-buffered saline (PBS), isoflurane, MMP3, and/or NNGH; treatment was administered one hour after TEER was initiated. (b, d) Endothelial barrier integrity was evaluated by calculating the mean TEER. ^∗∗∗∗^*p* < 0.0001, ^####^*p* < 0.0001. (e, f) WT or MMP3-KO (KO) BMVECs were grown with primary mouse astrocytes in Transwell chambers and treated with PBS, LPS, LPS, and NNGH, or MMP3, as indicated; then, the chambers were suspended in the wells of a 6-well plate, (e) red (40KD) or (f) blue (3KD) FITC-dextran was added to the chamber, and permeability was evaluated 24 hours later by measuring the intensity of dextran fluorescence in the plate wells. ^∗∗∗∗^*p* < 0.0001, ^∗∗∗^*p* < 0.001, ^∗∗^*p* < 0.010, ^####^*p* < 0.0001. Quantified data are summarized for 5 independent experiments.

The potential role of MMP3 in BBB permeability was also evaluated *in vitro* by using an electrical cell-substrate impedance sensing system (ECIS 8W10E+ array) to measure transendothelial electrical resistance (TEER) in monolayers of MMP3-KO or WT BMVECs. Because resistance measurements are inversely correlated with membrane permeability, the significantly lower measurements observed in isoflurane-treated (30 *μ*L/mL) WT cells than in WT cells that had been treated with saline indicated that isoflurane mildly increased BMVEC permeability. Resistance measurements in MMP3-KO BMVECs also declined in response to isoflurane treatment, but not significantly, and measurements were significantly greater in MMP3-KO BMVECs than in WT BMVECs regardless of treatment (Figures 5(a) and 5(b)). Furthermore, resistance measurements in both saline- and isoflurane-treated WT BMVECs declined significantly when the cells were cotreated with activated MMP3 (150 ng/mL) and increased significantly when isoflurane-treated WT BMVECs were cotreated with the MMP3 inhibitor N-isobutyl-N-(4- methoxyphenylsulfonyl)-glycyl hydroxamic acid (NNGH; 25 *μ*M) (Figures 5(c) and 5(d)).

To more accurately mimic *in vivo* conditions, permeability assessments were also conducted with cocultures of freshly isolated mouse BMVECs and primary mouse astrocytes. The cells were grown in Transwell chambers, and the chambers were suspended in the wells of a 6-well plate; then, FITC-dextran (Dextran-blue-3KD and Dextran-red-40KD) was added to the Transwell chamber, and permeability was evaluated by measuring the intensity of dextran fluorescence in the plate wells. Measurements were significantly lower in cocultures with MMP3-KO BMVECs than in cocultures with WT BMVECs and increased significantly in WT BMVEC cocultures after treatment with MMP3. Furthermore, measurements in both WT and MMP3-KO BMVEC cocultures increased significantly after treatment with lipopolysaccharide (LPS; 100 *μ*g/mL), which stimulates MMP3 activity and disrupts the BBB, but remained significantly lower in LPS-treated MMP3-KO cocultures than in LPS-treated WT cocultures, and the LPS-induced increase in permeability observed in WT cocultures was abolished by cotreatment with NNGH (Figures 5(e) and 5(f)). Collectively, the results from both our TEER and Transwell assays suggest that MMP3 increases BMVEC permeability and are consistent with our observations in MMP3-KO, WT, and WT+MMP3 mice.

### 3.4. MMP3 Regulates the Abundance of TJ Proteins and Adhesion Molecules by Modulating ERK Pathway Activity

**Figure 6:**
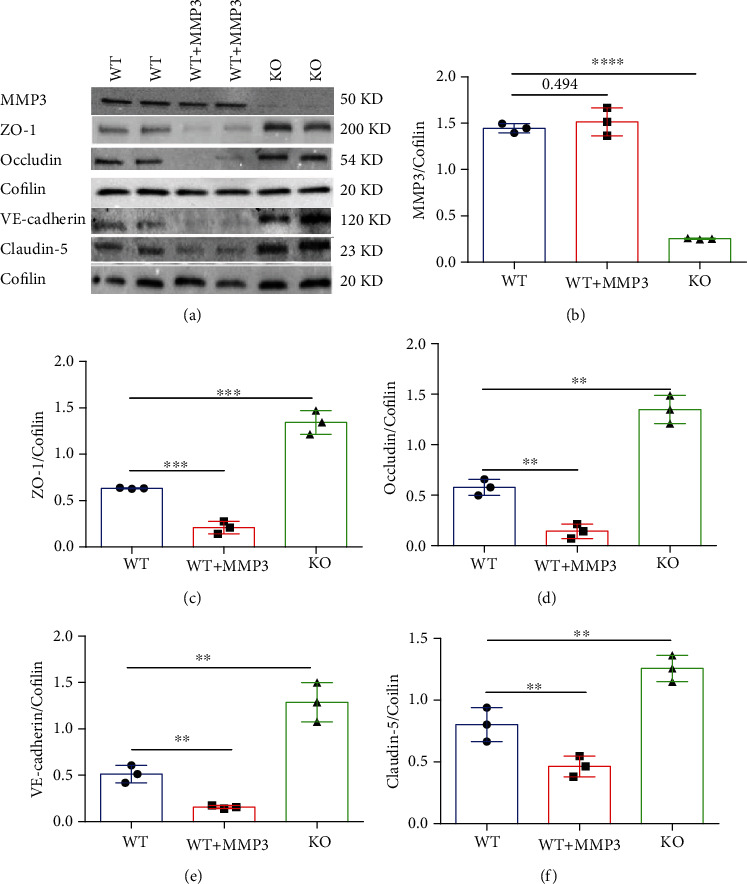
MMP3 regulates the abundance of junction proteins in BMVECs. (a–f) The abundance of MMP3, ZO-1, Occludin, VE-cadherin, and Claudin 5 were (a) evaluated via Western blot in WT BMVECs, WT BMVECs that had been treated with MMP3 (WT+MMP3), and MMP3-KO BMVECs (KO). Cofilin abundance was also evaluated to confirm equal loading, and then (b) MMP3, (c) ZO-1, (d) Occludin, (e) VE-cadherin, and (f) Claudin 5 measurements were quantified via normalization to Cofilin. ^∗∗∗∗^*p* < 0.0001, ∗∗∗*p* < 0.001, ^∗∗^*p* < 0.010. (g) WT, WT+MMP3, and MMP3-KO BMVECs were incubated with ZO-1 primary antibodies and fluorescent secondary antibodies (bar = 20 *μ*m), nuclei were counterstained with DAPI, and then (h) ZO-1 abundance was quantified via measurements of fluorescence intensity. ^∗∗∗∗^*p* < 0.0001. Quantified data are summarized for 3 experiments.

The selective permeability of the BBB is crucially dependent on the formation of tight junctions (TJs) and adherens junctions (AJs) [38]; thus, we investigated whether MMP3 may increase BBB permeability by disrupting expression of the TJ proteins ZO-1, occludin, and claudin-5, as well as the AJ protein vascular endothelial (VE)-cadherin; experiments were conducted with primary BMVECs isolated from the brains of MMP3-KO mice or their WT littermates. Notably, treatment with activated MMP3 did not change MMP3 expression in WT BMVECs; nevertheless, the expression of all four junction proteins was significantly lower in MMP3-treated WT BMVECs and significantly greater in MMP3-KO BMVECs, than in WT BMVECs without MMP3 treatment (Figures 6(a)–6(f)). ZO-1 expression was also evaluated via immunofluorescence in BMVECs that had been incubated with primary ZO-1 antibodies and fluorescent secondary antibodies (Figure 6(g)): measurements of fluorescence intensity declined significantly in WT cells after treatment with MMP3 and was significantly greater in MMP3-KO cells than in WT cells without MMP3 treatment (Figure 6(h)).

**Figure 7:**
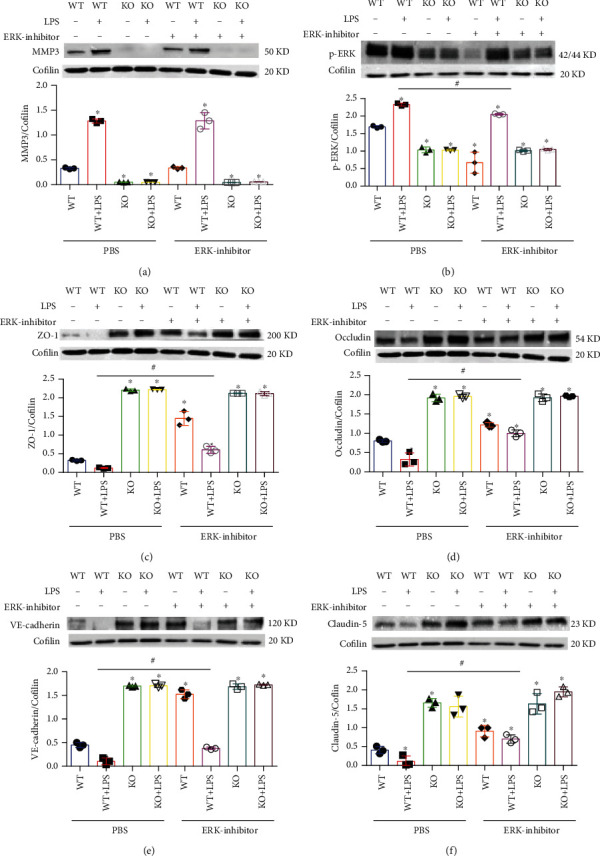
MMP3 regulation of BMVEC junction proteins is mediated by ERK. WT and MMP3-KO (KO) BMVECs were treated with LPS, saline, and/or an ERK inhibitor as indicated; then (a) MMP3, (b) phosphorylated ERK (p-ERK), (c) ZO-1, (d) Occludin, (e) VE-cadherin, and (f) Claudin 5 abundance were evaluated via Western blot and normalized to Cofilin abundance (^∗^*p* < 0.001 vs. WT+PBS in a; ^∗^*p* < 0.010 vs. WT+PBS, ^#^*p* < 0.010 for all other panels (Please verify)). Quantified data are summarized for 3 experiments.

The ERK1/2 pathway induces BBB hyperpermeability by changing the composition of TJs [15, 16]; thus, we investigated whether ERK has a role in MMP3-regulated TJ protein expression by comparing measurements in BMVECs treated with saline or an ERK inhibitor. Experiments were conducted with both WT and MMP3-KO BMVECs, and since the expression of MMP3 in WT BMVECs did not change when the cells were treated with recombinant MMP3, MMP3 expression was upregulated via treatment with LPS (100 *μ*g/mL). LPS induced ERK phosphorylation in BMVECs, and induction was prevented by either MMP3 KO or the use of an ERK inhibitor. p-ERK abundance was also lower in all MMP3 KO groups than in the WT group, and in WT BMVECs, ERK inhibition did not alter MMP3 expression either with or without concomitant LPS treatment, but measures of TJ protein expression were significantly higher in cells treated with the ERK inhibitor alone or cotreated with the ERK inhibitor and LPS than in the saline-alone or saline-LPS treatment groups, respectively. Neither ERK inhibition nor LPS treatment altered MMP3 expression, ERK activation, or TJ protein expression in MMP3-KO BMVECs, but TJ protein levels were significantly higher than in WT BMVECs (Figures 7(a)–7(f)). Thus, MMP3 appears to reduce the integrity of BMVEC monolayers, at least in part, by upregulating ERK signaling, which subsequently reduces the abundance of junction proteins.

## 4. Discussion

**Figure 8:**
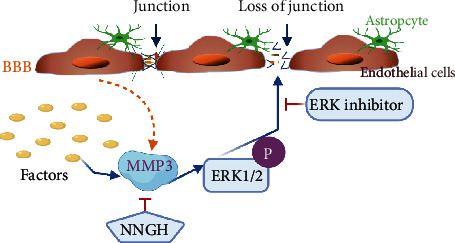
Schematic model for MMP3 regulation of BBB permeability. MMP3 is produced by BMVECs in response to external factors and phosphorylates ERK, which subsequently disrupts tight junctions between adjacent ECs, thereby increasing BBB permeability. BBB integrity can be preserved via ERK inhibition or by blocking MMP3 activity with NNGH.

To the best of our knowledge, the study presented here is the first to demonstrate that MMP3 deficiencies suppress, while treatment with activated MMP3 enhances, isoflurane anesthesia in mice. BBB permeability was also quantified in mouse brains via the extravasation of intravenously administered dyes (Evans blue and sodium FITC) and in monolayers of BMVECs via TEER and Transwell assays: barrier integrity was significantly greater in the brains of MMP3-KO mice and in monolayers of MMP3-KO BMVECs than in WT mice and WT BMVEC monolayers, respectively, whereas MMP3 upregulation reduced BBB integrity *in vivo* and the integrity of BMVEC monolayers *in vitro*. Notably, isoflurane significantly reduced TEER assessments of integrity in monolayers of WT BMVECs, but not MMP-KO BMVECs, and treatment with MMP3 exaggerated isoflurane-induced BMVEC barrier dysfunction, but not in the presence of NNGH, an MMP3 inhibitor, which suggests that the BBB permeability of isoflurane may also increase in an MMP3-dependent manner. Furthermore, MMP3 activated ERK signaling, and MMP levels were negatively correlated with the abundance of TJ and AJ proteins in BMVECs, but not when the cells were cotreated with an ERK inhibitor. Taken together, the results from our experiments with WT and MMP3-KO mice and BMVECs suggest that MMP3 increases BBB permeability and that this effect may be at least partially mediated by increases in ERK pathway activity and the ERK-induced disruption of inter-EC junctions (Figure 8).

*In vivo* assessments of MMP activity are technically challenging, which may partially explain why the results from previous investigations of MMPs in endothelial barrier function have been limited and somewhat inconsistent. For example, both MMP2 and MMP9 downregulated ZO-1 expression and enhanced BBB permeability in response to focal cerebral ischemia-reperfusion injury [39], but MMP9 deficiency did not reduce disruption of the BBB during viral encephalomyelitis [40]. The role of MMP3 in BBB maintenance is even less well-studied, but our observation that MMP3 increases BBB permeability by reducing the expression of several junction proteins, including ZO-1, claudin-5, and occludin, is consistent with a previous report that MMP3 disrupts the blood-spinal cord barrier [41]. Furthermore, ERK signaling appears to regulate TJ protein expression in the proximal epididymis of mice [42], and the results reported here indicate that MMP3-induced declines in TJ protein levels are strongly dependent on ERK activation: levels of ERK phosphorylation correlated positively with MMP3 activity and negatively with TJ protein levels, and ERK inhibition increased TJ protein levels in WT BMVEC monolayers, but not in monolayers of MMP3-KO BMVECs. The mechanism that links ERK phosphorylation to the disruption of BBB junctional proteins is unknown but could involve the ubiquitin ligase complex, which targets phosphorylated proteins for degradation [43]; these topics will be addressed in future studies.

MMPs are essential for normal brain function, but elevated levels of MMP expression are believed to contribute to pathological CNS conditions by damaging the neurovascular unit and disrupting the BBB, perhaps via the degradation of TJ and basement-membrane proteins [13, 44]. Oxidative stress is also implicated in activation of MMPs and impaired BBB [45]. Several studies have suggested that MMP2 and MMP9 may contribute to BBB breakdown [13, 17]—for example, MMP9 appears to mediate the loss of TJ stability observed in mice with 1,2-dichloroethane-induced brain edema [46]—and our observation that MMP3 mRNA levels were much higher in BMVECs than in MVECs from other organs (e.g., the heart, lungs, spleen, and kidney) suggests that it may have a unique and essential role in BMVECs, particularly since many of the ECM proteins that form the basal lamina of cerebral blood vessels can be proteolysed by MMP3 [19]. Isoflurane has also been linked to BBB disruption [47, 48], but may protect against acute lung injury by maintaining endothelial barrier integrity [11], so the effect of isoflurane on BBB permeability remains somewhat unclear.

One of the significant findings of this study is that MMP3 was associated with elevated anesthetic sensitivity and BBB opening. Because the BBB is the primary regulator of exchange between the peripheral circulation and the brain, and is the key surface through which systemically administered drugs access the CNS [8], the complexity of the BBB provides many unique opportunities for drug delivery. Thus, our results suggest that MMP3 may be a useful adjunctive treatment for enhancing the efficacy of other neurotherapeutics [49], which could reduce the occurrence and/or severity of side effects (as well as cost) by enabling patients to be treated with lower doses of the primary treatment [35, 50]. However, increases in BBB permeability could also enable blood-borne immune cells to enter the brain and provoke a neuroinflammatory response [51], as illustrated by growing evidence that BBB disruption is associated with brain inflammatory conditions such as Alzheimer's disease and multiple sclerosis [52]. Oxidative stress is also implicated in MMP activation and blood-brain barrier injury [53]. However, despite enormous efforts, CNS drug discovery still relies on improving the BBB, and the success of CNS therapeutic development depends on techniques for modulating BBB permeability and the kinetics of drug distribution to ensure optimal CNS penetration [35].

Unlike other MMPs, MMP3 is predominantly expressed in ECs, and our results indicate that it is more highly expressed in the brain than in other organs; nevertheless, our *in vivo* experiments were conducted with global MMP3-KO mice, so our observations in these animals could have been influenced by the loss of MMP3 expression in non-ECs, as well as BMVECs. Furthermore, since direct MMP3 administration did not increase MMP3 levels in BMVECs, LPS was used to upregulate MMP3 expression in our in vitro studies and, consequently, we cannot exclude a potentially MMP3-independent role for LPS in TJ stability. Future studies will address these limitations by conducting experiments in EC-specific MMP3-KO mice, by investigating the use of other MMP3 inhibitors, and by determining how LPS regulates MMP3 expression and BBB integrity.

## 5. Conclusions

In summary, the results presented here are the first to reveal the function of MMP3 in the BBB and suggest that it has an essential role in the brain microvasculature that differs from its function in other vessels. We have shown that MMP3 increases BBB permeability by upregulating the ERK signaling pathway, which subsequently reduces TJ and AJ protein abundance in BMVECs. Oxidative stress often leads to impairment of BBB. Since the BBB is the primary regulator of exchange between the peripheral blood and the brain, our observations likely have important implications for treating neuroinflammatory conditions and other CNS disorders involving the endothelial MMP3 pathway.

## Data Availability

Data are available on request.

## Supplementary Materials

Supplementary Materials Primary culture and identification of astrocytes. Primary culture and identification of BMVECs. Supplementary results: identification and morphology of primary cultured astrocytes and BMVECs. Supplementary Figure 1.
