# Supplementary material for: Increase in Blood-Brain Barrier (BBB) Permeability Is Regulated by MMP3 via the ERK Signaling Pathway
Source: Oxid Med Cell Longev. 2021 Mar 30;2021:6655122. doi: 10.1155/2021/6655122 (PMC8026308; doi:10.1155/2021/6655122)
Supplement: Supplementary Materials — Primary culture and identification of astrocytes. Primary culture and identification of BMVECs. Supplementary results: identification and morphology of primary cultured astrocytes and BMVECs. Supplementary Figure 1. [file 6655122.f1.zip › 6655122.f1/Supplementary material (1).pdf]

Isolation and identification of mice brain microvascular endothelial cells (BMVECs) and astrocytes

Supplementary Methods

Primary culture and identification of astrocytes

Two mice were decapitated and disinfected in 75% ethanol for 3–5 minutes. The whole brains were dissected and placed into pre-cooled D-Hanks' solution. Next, the meninges and blood vessels were carefully removed while the brains were on ice. The cerebral cortex was separated and washed three times in D-Hanks' solution before being cut into blocks (1mm^3) in Dulbecco's Modified Eagle's Medium (DMEM; Gibco, Grand Island, NY, USA). After this, the tissue was digested in 0.25% trypsin (Sigma, St. Louis, MO, USA) and filtered through a 200-mesh screen. The filtrate was centrifuged at 140g for 10 minutes and the supernatant discarded. The cells were resuspended in DMEM and placed in a 75cm^2 disposable culture bottle. Afterward, the cell suspension was transferred to another bottle and cultured at 37°C and 5% CO2. Furthermore, the DMEM was replaced every three days, and the cells were observed under an inverted microscope. We were able to purify astrocytes nine days later. The cultured bottle was incubated at a constant temperature of 37°C, on a DHS rotating bed at 240 RPM. Finally, suspended cells were removed after 18 hours, and astrocytes were cultured in DMEM, 10% FBS, and antibiotics.

Primary culture and identification of BMVECs

8 weeks-old mice were used for isolation of the BMVECs. Briefly, the brains were isolated, and gray matter was carefully dissected, minced in Hank's balanced salt solution (HBSS; Invitrogen Corp, CA, USA), and gently homogenized in a Dounce homogenizer. Next, the microvessels were separated from erythrocytes by using a Percoll gradient (GE Healthcare Bio-Sciences, PA, USA). The microvessels were then digested in a solution of 1mg/mL of collagenase (Roche, IN, USA) for 30 minutes at 37°C in HBSS. Primary ECs were grown on plates coated with type IV collagen (BD Bioscience, NJ, USA) in Endothelial Cell Medium (ECM) purchased from ScienCell Research Laboratories (Carlsbad, CA USA) at 37°C in a humid atmosphere with 5% CO_2. To obtain a 99%

pure culture, we purified ECs with puromycin for 2 days. Finally, the BMVECs were cultured in DMEM, heparin (0.1mg/ml), 1mg/ml ECGF (0.15mg/ml), 20% FBS, and antibiotics.

Supplementary Results

Identification and Morphology of primary cultured astrocytes

Most primary cultured astrocytes had adhered to the wall by the 7th day of culture, and the number of cell bodies had markedly increased, with many slender branching processes stretching between neighboring cells seen (supplemental figure 3A). After immunofluorescence staining for GFAP and MMP3, fluoresced green staining demonstrated distinct cellular contours and WT astrocytes (Supplemental figure 1B and 1D).

Identification and Morphology of primary cultured BMVECs

Both MMP3 mRNA and protein levels in BMVECs derived from KO mice were much lower than in WT mice ($p<0.001$; supplemental figures 2A and 2B). By day 14, the BMVECs had integrated, forming a dense, cobblestone-like monolayer (supplemental figure 1A). After immunofluorescence staining for vWF and MMP3, fluoresced green staining was observed in BMVECs (supplemental figure 1C and 1E)

Supplemental figure legend:

Supplemental figure 1. Identification of the BBB model. A. Image of endothelial cells and astrocytes. B. GFAP immunofluorescence staining of Astrocyte. C. VWF immunofluorescence staining of the mice BMVECs. D. MMP3 immunofluorescence staining of astrocyte. E. MMP3 immunofluorescence staining of BMVECs.

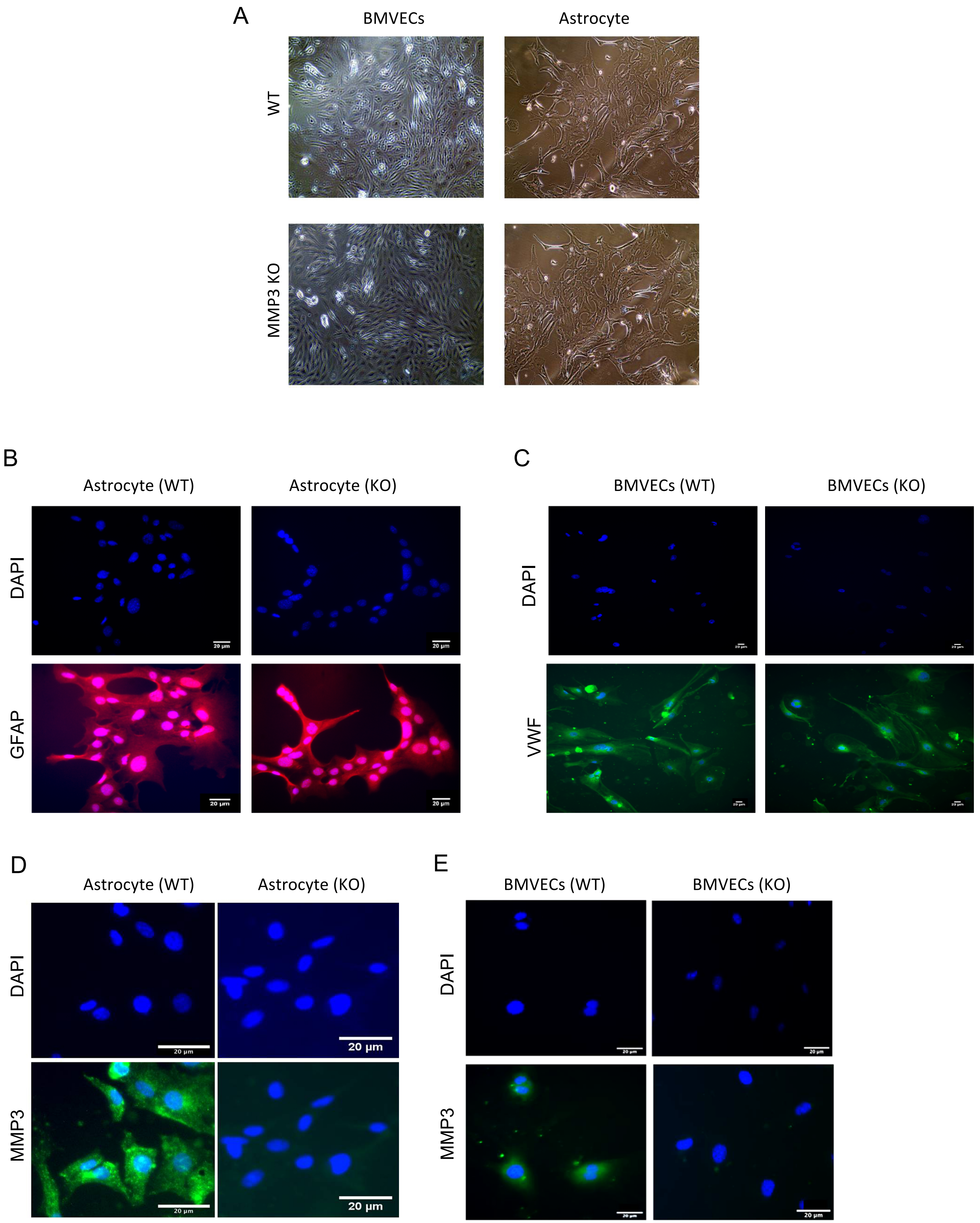

Supplemental figure 1
