# Supplementary figures and images for: Increase in Blood-Brain Barrier (BBB) Permeability Is Regulated by MMP3 via the ERK Signaling Pathway
Source: Oxid Med Cell Longev. 2021 Mar 30;2021:6655122. doi: 10.1155/2021/6655122 (PMC8026308; doi:10.1155/2021/6655122)

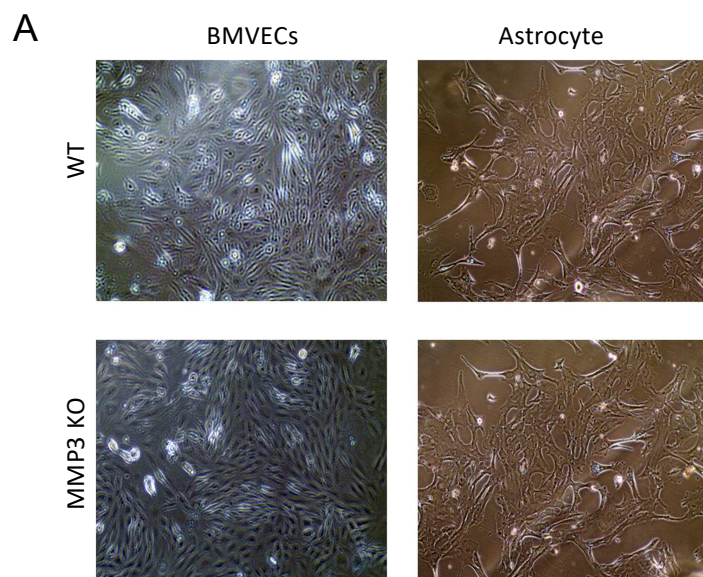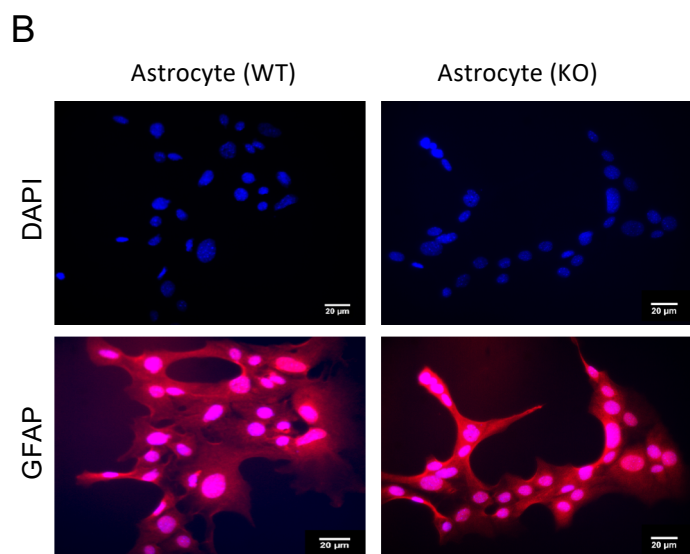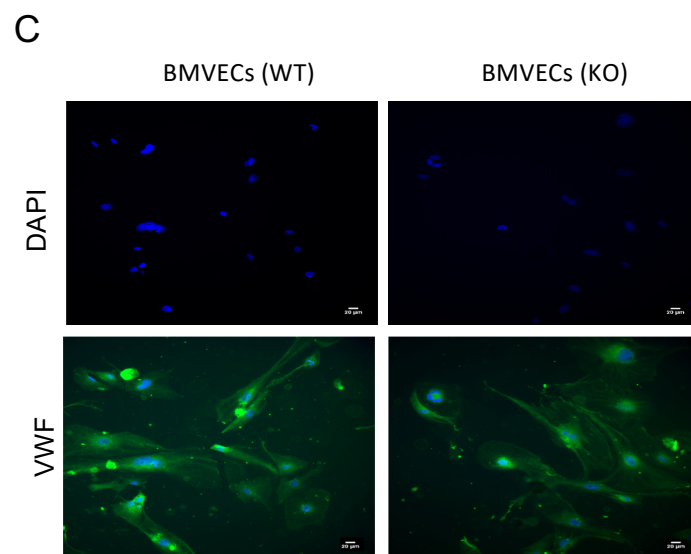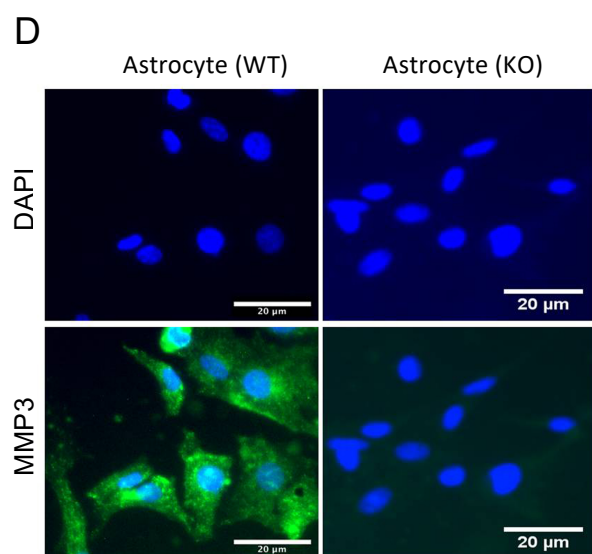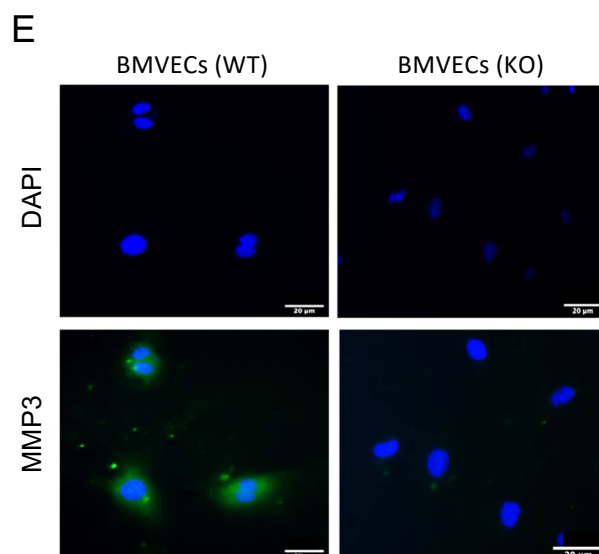

Supplemental figure 1

Supplement: Supplementary Materials — Primary culture and identification of astrocytes. Primary culture and identification of BMVECs. Supplementary results: identification and morphology of primary cultured astrocytes and BMVECs. Supplementary Figure 1. [file 6655122.f1.zip › 6655122.f1/Supplemental figure 1.pdf]
